# Unearthing the invisible: simulating maize root domestication through deep time

**DOI:** 10.1111/nph.70376

**Published:** 2025-07-04

**Authors:** Peng Yu

**Affiliations:** ^1^ Plant Genetics, TUM School of Life Sciences Technical University of Munich 85354 Freising Germany; ^2^ Emmy Noether Group Root Functional Biology, Institute of Crop Science and Resource Conservation University of Bonn 53117 Bonn Germany

**Keywords:** domestication, genetics, maize, root system architecture, seminal roots

## Abstract

This article is a Commentary on Lopez‐Valdivia *et al*. (2025), **248**: 339–353.

The domestication of maize is a cornerstone in the history of agriculture, yet much of the evolutionary narrative has focused aboveground – ears, leaves, and stems (Hake & Ross‐Ibarra, 2015). To meet the food demands of the twenty‐first century, plant scientists must look belowground, as the yield gains of the twentieth century were largely driven by fertilizers and aboveground plant improvements (Bishopp & Lynch, 2015). Root systems are vital for plant growth and global food security, as they extract water and minerals from the soil to support aboveground functions. In maize, the fibrous root system comprises embryonic roots like the primary and seminal roots – critical for early seedling vigor and nutrient uptake – and postembryonic shoot‐borne roots, such as crown and brace roots that dominate at later stages, providing anchorage and plasticity for efficient resource foraging (Hochholdinger, 2009). Lateral roots and root hairs further enhance soil exploration and nutrient acquisition (Yu *et al*., 2016), while the cellular organization within each root type and spatial arrangement among diverse root types contribute significantly to the adaptability and resilience of maize under harsh, variable environmental conditions (Lynch, 2022). Genetic studies in maize reveal that auxin‐centered networks, involving key genes, such as *ROOTLESS CONCERNING CROWN AND SEMINAL ROOTS* (*RTCS*) and *ROOTLESS WITH UNDETECTABLE MERISTEMS 1* (*RUM1*), regulate the development of different root types – including shoot‐borne, seminal, and lateral roots – by coordinating their initiation and growth (Hochholdinger *et al*., 2018). Building on this genetic and developmental understanding of maize root architecture, in an article published in this issue of *New Phytologist*, Lopez‐Valdivia *et al*. (2025; pp. 339–353) venture underground to trace how root phenotypes may have evolved in response to anthropogenic and environmental challenges over the last 18 000 yr in the Tehuacán Valley, a cradle of early maize cultivation. By integrating paleoclimatic data, ancient DNA, and high‐resolution functional‐structural modeling, the authors reconstruct the hidden evolutionary dynamics of maize root systems with a level of temporal and ecological precision rarely achieved in domestication research.

In silico *models have long promised insight into evolutionary questions inaccessible to experimental study. Here, the authors deliver on that promise*.

## Bridging the past and present through simulation

The core of Lopez‐Valdivia's study lies in *OpenSimRoot* (Postma *et al*., 2017), a process‐based, spatially explicit model capable of simulating interactions between plant root systems and their soil–atmosphere environments. By incorporating transitions in climate, soil fertility, and irrigation practices, the authors convincingly simulate the selective pressures that may have favored three key root traits: reduced nodal root number (NRN), multiseriate cortical sclerenchyma (MCS), and increased seminal root number (SRN). *In silico* models have long promised insight into evolutionary questions inaccessible to experimental study. Here, the authors deliver on that promise. Their model suggests that reduced NRN and MCS first emerged *c*. 8000 yr before present (yBP), a time of rising atmospheric CO_2_ and declining soil fertility due to the early adoption of cultivation. Increased SRN, by contrast, appears more recently (*c*. 3500 yBP), coinciding with intensified agriculture and demographic expansion in the region. These findings lend quantitative support to the hypothesis that root evolution was shaped not only by environmental constraints (Yu *et al*., 2024), but also by human‐driven ecological change. The shift of nitrogen availability from topsoil to subsoil – a consequence of irrigation and erosion – likely drove the evolution of deeper root systems (Saengwilai *et al*., 2014). These insights open new avenues for future research that integrates ecological modeling and functional genomics to experimentally validate how specific environmental transitions and cultural innovations may have orchestrated the sequential selection of root traits during maize evolution.

## Paleogenomics as a rooted narrative

A striking contribution of this study is its use of ancient maize DNA to map changes in allele frequencies associated with root phenotypes. By integrating allelic variation at loci, such as BIG EMBRYO1 and RUM1 across a time series of archeological maize samples, the authors provide evidence for a delayed genetic shift toward modern SRN relative to NRN and MCS. This delay is not merely a curiosity – it aligns with archeological records of increased agricultural intensification and population density, suggesting a strong link between cultural development and biological adaptation. Moreover, the model's ability to predict the impact of individual and combined root traits across a dynamic environmental gradient adds significant explanatory power. The authors go beyond correlative narratives, showing how combinations of traits (e.g. reduced NRN and MCS) conferred greater fitness in specific temporal windows, revealing potential evolutionary ‘sweet spots’ in phenotype–environment interactions. In parallel, using a Multiparent Advanced Generation Inter‐Cross population representing a latitudinal cline in SRN in Mexican maize, Yu *et al*. (2024) identified key genomic regions – including the *RTCS* gene, which is crucial for seminal and crown root development – and developed a genome‐wide predictive model that captured the latitudinal SRN trend, suggesting directional selection on RTCS and other loci for local adaptation. These coherent studies (Lopez‐Valdivia *et al*., 2022, 2025; Yu *et al*., 2024) that integrated root genetics with their ecological roles in shaping root phenotypes are crucial for understanding how domestication and environmental adaptation have driven maize evolution and improved crop resilience.

## Challenging the assumptions of seed size and SRN

Lopez‐Valdivia *et al*. thoughtfully explore the nuanced relationship between seed size and seminal root number, suggesting that, rather than being entirely genetically independent as some recent studies propose, seed carbohydrate reserves may have subtly influenced SRN expression during the early stages of domestication. Indeed, domestication‐driven increases in seed size enabled maize to develop more seminal roots than its wild ancestor teosinte, enhancing early nitrogen and phosphorus uptake and improving nutrient acquisition under variable environmental conditions (Perkins & Lynch, 2021). While seed size alone may not dictate SRN in modern germplasm (Yu *et al*., 2024), its role in the teosinte‐to‐maize transition warrants deeper investigation, particularly through functional studies in a broader panel, including extant teosintes (Lopez‐Valdivia *et al*., 2025). In fact, sugar maize germplasm produces significantly fewer seminal roots than other modern types; however, analyses of near‐isogenic lines carrying endosperm sugar mutants (such as *sugary1* and *shrunken2*) reveal that seminal root formation is independent of carbohydrate availability during seed development (Yu *et al*., 2024), suggesting that the sugar metabolic pathway may influence embryonic root development through more complex, regulatory mechanisms. A promising direction for future research is to investigate how specific types and spatial distributions of sugars – beyond overall carbohydrate availability – act as signaling molecules or developmental cues influencing root initiation and patterning, particularly in embryonic root formation; integrating sugar transport, sensing, and hormonal crosstalk may reveal novel regulatory networks underlying genotype‐specific root architecture.

## Opportunities and broader implications

As with any modeling effort, simplifications are inevitable. The study does not incorporate flowering time, biotic interactions, or the possible effects of microbial symbioses, such as mycorrhizas (Galindo‐Castañeda *et al*., 2019) and plant growth promoting bacteria (Yu *et al*., 2021; He *et al*., 2024) – factors that likely influenced root development and functioning. Furthermore, the assumption that greater biomass correlates directly with evolutionary fitness overlooks the potential roles of reproductive success and competitive interactions in shaping root traits. Nonetheless, the heuristic nature of *OpenSimRoot* does not detract from its value here. Rather, it underscores the importance of models as hypothesis‐generating tools, especially in domains where experimental replication of past environments is impossible. The ability to test counterfactual scenarios – what if MCS appeared before reduced NRN, or if irrigation emerged earlier – is particularly powerful.

This work offers a template for future studies aiming to integrate paleogenomics, archeology, and functional modeling. It challenges researchers to consider root traits as dynamic and responsive to anthropogenic change – traits not merely inherited but co‐evolved with agriculture. Importantly, it provides a roadmap for how such insights might guide breeding strategies in the face of climate change, where deep rooting and nutrient foraging efficiency may become increasingly valuable. Moreover, the findings open questions about convergent root evolution in other domesticated cereals. Did similar trait transitions occur in wheat, barley, or sorghum in response to early irrigation and soil degradation? Can *in silico* modeling be expanded to other centers of domestication?

Overall, Lopez‐Valdivia *et al*. offer a bold and data‐rich reconstruction of maize root evolution across millennia. By combining ancient DNA, paleoclimate reconstructions, and cutting‐edge simulation, they shift the narrative of domestication from the visible to the subterranean. Their study not only illuminates how roots adapted to ancient human landscapes – it roots maize more deeply in its evolutionary and cultural past.

## Disclaimer

The New Phytologist Foundation remains neutral with regard to jurisdictional claims in maps and in any institutional affiliations.
